# Immune Signatures and Survival of Patients With Metastatic Melanoma, Renal Cancer, and Breast Cancer

**DOI:** 10.3389/fimmu.2020.01152

**Published:** 2020-06-09

**Authors:** Kilian Wistuba-Hamprecht, Cécile Gouttefangeas, Benjamin Weide, Graham Pawelec

**Affiliations:** ^1^Division of Dermatooncology, Department of Dermatology, University Medical Centre Tübingen, Tübingen, Germany; ^2^Immunoguiding Workgroup of the Cancer Immunotherapy Association (CIP/CIMT), Mainz, Germany; ^3^Department of Immunology, Institute for Cell Biology, University of Tübingen, Tübingen, Germany; ^4^Germany and German Cancer Consortium (DKTK) and German Cancer Research Center (DKFZ) Partner Site Tübingen, Tübingen, Germany; ^5^Cluster of Excellence iFIT (EXC2180) “Image-Guided and Functionally Instructed Tumor Therapies”, University of Tübingen, Tübingen, Germany; ^6^Health Sciences North Research Institute, Sudbury, ON, Canada

**Keywords:** immune signatures, biomarker, melanoma, renal cancer, breast cancer

## Abstract

Despite remarkable recent progress in treating solid cancers, especially the success of immunomodulatory antibody therapies for numerous different cancer types, it remains the case that many patients fail to respond to treatment. It is therefore of immense importance to identify biomarkers predicting clinical responses to treatment and patient survival, which would not only assist in targeting treatments to patients most likely to benefit, but might also provide mechanistic insights into the reasons for success or failure of the therapy. Several peripheral blood or tumor tissue diagnostic and predictive biomarkers known to be informative for cancer patient survival may be applicable for this purpose. The use of peripheral blood (“liquid biopsy”) offers numerous advantages not only for predicting treatment responses at baseline but also for monitoring patients on-therapy. Assessment of the tumor microenvironment and infiltrating immune cells also delivers important information on cancer-host interactions but the requirement for tumor tissues makes this more challenging, especially for monitoring sequential changes in the individual patient. In this contribution, we will review our findings on immune signatures potentially informative for clinical outcome in melanoma, breast cancer and renal cell carcinoma, particularly the outcome of checkpoint blockade, by applying multiparametric flow cytometry and mass cytometry, routine clinical monitoring and functional testing for predicting and following individual patient responses to therapy.

## Introduction

The long-standing controversy as to whether the immune system performs immunosurveillance against cancer, as originally proposed by Burnet (1), and the accompanying skepticism as to whether immune-based treatments would ever be effective (2) was finally laid to rest with the development of clinically effective immunomodulatory antibody treatments [immune checkpoint inhibition, ICI (3)], culminating in the Nobel Prize for Physiology or Medicine in 2018. Nonetheless, there are countless reasons why some cancer patients may not respond at all, or later become refractory to ICI, almost matched by the large number of published papers discussing this issue (4). For routine application and selection of the best therapy with the least cost and fewest side-effects, a major unmet need is to define robust biomarkers predicting meaningful response. These would ideally be as simple as possible and predict the likelihood of response not only prior to but also during therapy. For the purpose of monitoring response to therapy, and for ease of application in routine clinical settings, biomarkers established from a small sample of peripheral blood would offer many advantages over tissue biopsy. Parameters measurable in peripheral blood mononuclear cells (PBMC) include antigen presentation capacity, T cell antigen-specificity, activation and differentiation/activation states, cytokine and chemokine production, quantity and quality of regulatory T cells (Tregs) and of so-called myeloid-derived suppressor cells (MDSCs), as well as circulating cancer cells themselves, cell-free DNA and exosomes from the tumor. What would be more difficult but theoretically not impossible to determine using blood would be the presence of tumor-associated antigens and MHC expression on the cancer cells, their mutational burden and neoantigen landscape, the expression of cell membrane ligands directly involved in the regulation of T cell function, as well as more mundane parameters such as tumor burden. Although tumor tissue is certainly highly informative when searching for such immune biomarkers, one evident limitation is that these are rarely available for all patients and at different times during therapy. Hence, peripheral blood, which can be repeatedly obtained during therapy in a minimally invasive manner, is an attractive alternative, despite not representing the place “where the action is.” Here we summarize predominantly our own work on constellations of peripheral biomarkers informative for responses to ICI (mostly anti-CTLA-4 or anti-PD-1 in melanoma). We contrast these with tumor-infiltrating immune cells (TIICs) in breast and kidney cancers where comparisons between peripheral and tissue data are more readily possible. The overall aim of the work reviewed here was to generate minimal clusters of the simplest possible biomarkers with maximal predictive ability for routine application in the clinic (Figure 1).

**Figure 1 F1:**
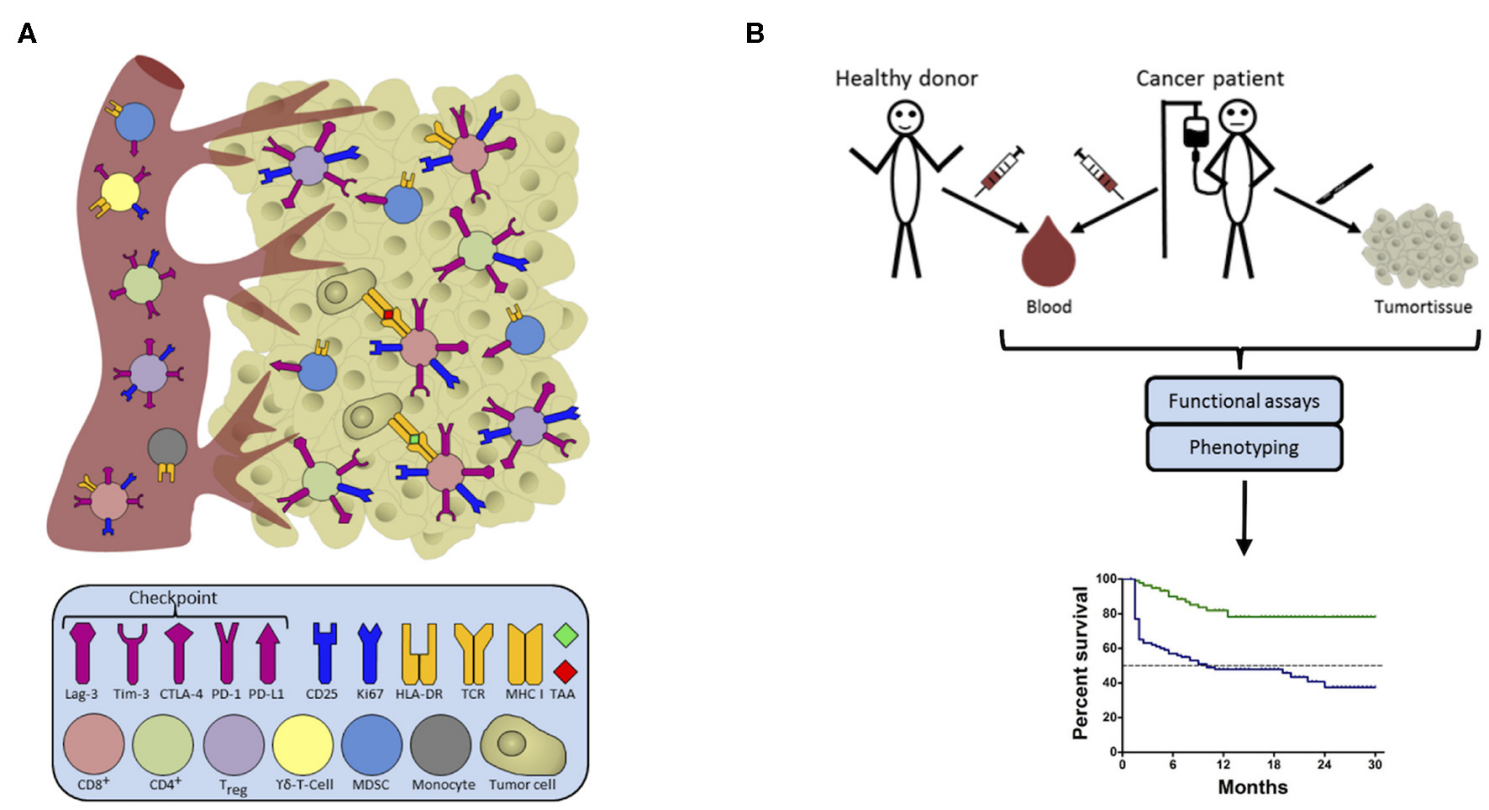
Candidate biomarkers in the host-cancer/cancer-host interaction. **(A)** Intra-tumoral leucocytes commonly consist of a highly diverse pool of cells which may allow prognostic or even predictive associations with the course of disease/treatment outcome. Some of these cells involved in cancer immunosurveillance migrate between tissues and can thus also be detected in peripheral blood. The figure shows cells in the blood on the left, and in the tumor on the right, color-coded to represent the different cells involved, along with their surface receptors. **(B)** Blood is an ideal source of material for the determination of clinically relevant biomarkers as it is easy to access repeatedly, and allows comparison with healthy donors. Functional assays combined with phenotyping provide constellations of immune parameters constituting an immune signature with a closer correlation with survival than any single factor. From a practical point of view, we should aim to replace functional assays by rapid *ex vivo* phenotyping approaches to pave the way for defining novel biomarkers for use in a routine clinical setting.

## Peripheral Biomarkers for Melanoma Assessed as *in vitro* T Cell Responses to Tumor-Associated Antigens

With the above in mind, our interest in establishing immunological biomarkers informative for survival of patients with metastatic melanoma predated the introduction of ICI and stemmed from early studies on melanoma patients surviving for an unusually prolonged time on conventional therapy or other non-classical therapies. At that time, we undertook a small RNA vaccination study that sought to immunize individual melanoma patients with personalized mixtures of shared cancer testis and lineage antigens identified as expressed by the resected tumor (5). These included NY-ESO-1, Melan-A, MAGE-A3 and survivin as well as several others. We incubated pre-vaccination PBMCs from each patient with mixtures of overlapping peptides representing each entire molecule to which the patient would be vaccinated, and then restimulated with the same peptides thereafter. The assay readout was CD4+ and/or CD8+ T cell activation as assessed by simultaneous intracytoplasmic staining for 6 pro- and anti-inflammatory cytokines (IL 2, IFN-γ, TNF, IL 4, IL 5 or IL 10, and IL 17). Thus, this demanding assay system assesses the capacity of the immune cells in the individual patient's blood to pick up, process and present antigen by antigen-presenting cells (APC) in a manner triggering memory T cell activation and proliferation, and indicates whether the response is mediated by CD4+ or CD8+ T cells, and whether predominantly pro- or anti-inflammatory cytokines are produced, as well as revealing which potential tumor-associated antigens (TAA) can be recognized by the patient's T cells. This approach had first been successfully applied to document increasing frequencies of TAA-reactive CD8+ T cells in a patient responding to intra-lesional injection of IL 2 (6). Using this same assay, we next accessed our biobank of cryopreserved PBMCs from late-stage melanoma patients on conventional therapy and retrospectively associated responses to TAA by patients surviving for longer than usual (>2 years at that time), less than usual (<6 months) or in between. We found that although all patients' PBMCs responded to the positive control peptides (matrix protein and nucleoprotein peptides from influenza), the frequency of patients responding to NY-ESO-1 and/or Melan-A in the “long-survivor” group was significantly greater than in the “short-survivor” group. Patients responding to more than one TAA did better than those responding to none or only one. Interestingly, responses to NY-ESO-1 mediated by either CD4+ or CD8+ T cells were associated with longer survival, whereas CD8+ but not CD4+ T cell responses to Melan-A, were beneficial (7). Responses to two other TAA tested were not informative because almost all patients responded to MAGE-A3 and almost none to survivin (8). Prospective studies confirmed this association and went further to show that not only the identity of the antigen and responding T cell subset but also the nature of the T cell response against that antigen was informative for survival in these patients (7). In more recent independent studies, we have again observed predictive capacities of NY-ESO-1- and Melan-A-reactivities also for the outcome of melanoma patients under ICI with anti-PD-1 ± CTLA-4 antibodies (Zelba et al., personal communication) raising the question of potential advantages of T cells recognizing shared tumor antigens as one of several modules in future treatment strategies. Ongoing trials targeting in particular NY-ESO-1 might help to answer this question (for example NCT01967823, NCT03029273, NCT02775292).[Fig F1]

## Peripheral Biomarkers for Melanoma Assessed by Surface Marker Phenotyping of Immune Cells

A more conventional approach, easier to standardize and apply in routine clinical practice than the functional assays described above, monitors the presence of different immune cells in the peripheral blood by flow cytometry. To maximize data density from small blood samples, single cell, multi-parameter analysis has made great strides recently. In an early study, using a 38-channel time-of-flight mass cytometry (CyTOF) approach in 2013 we investigated the peripheral immune landscape (using PBMCs) in what was at the time the largest cohort of stage IV melanoma patients and age-matched healthy individuals subjected to this new technique (9). We compiled a detailed immune signature of T cells, NK cells, B cells and myeloid cells and their subsets and found that superior survival was characterized by relatively high proportions of differentiated NK-cells and a balanced distribution of monocytic MDSC (mMDSC)-like and APC-like phenotypes (HR: 0.2) (10). The predictive capacity of a comparable myeloid APC-like phenotype was reported by Krieg et al., in a similar high-dimensional CyTOF immunomonitoring study in melanoma under PD-1 blockade (11). Not only classical T cells, but also T cells carrying the alternative γδ T cell receptor can exert strong anti-tumor, but also under certain circumstances pro-tumor functions, as reviewed by others elsewhere (12). We suggest that these cells must also be considered when generating informative immune signatures because we found in a discovery study that low frequencies of Vδ1+ γδ T cells correlated with prolonged overall survival (OS) (13).

## Peripheral Biomarkers for Melanoma With Ipilimumab Treatment

For the purpose of clinical exploitation not relying on complex biological assays or specialist multi-parameter flow cytometry, simpler assays would be most useful and most likely to find widespread employment. As ipilimumab came into routine use as the first ICI agent licensed in 2011, we asked whether the cell surface immune signatures and intracellular FoxP3 staining would remain informative for patients receiving this agent, relative to conventional markers like LDH serum levels (14). We accessed our PBMC biobank from a large multi-center study to assess immune cell frequencies and clinical metadata before therapy start, in order to investigate potential correlations at the single and multiple factor level. We identified a model comprising a compound signature of low serum LDH-levels, absolute monocyte counts, mMDSC frequencies, high absolute eosinophil counts, Treg frequencies and relative lymphocyte counts associated significantly with a favorable outcome following ipilimumab treatment. For patients with a risk score of 0 in this model, the 2-year survival rate was 40.8%, whereas for those with a risk score ≤ 130 it was only 17.3%, and, strikingly, no patient with a risk score > 130 survived >15 months (15). Our data confirmed previous work reporting on the poor prognosis of patients with high LDH (16, 17), MDSC levels (17–19) or eosinophils (20) under ipilimumab.

In a follow up analysis of partially overlapping cohorts, we investigated changes of 22 factors (15 immune cell populations and seven routine blood counts) at two time points under therapy (2–8 and 8–14 weeks after start of ICI). We identified amongst others, significant increases in the expression of the proliferation marker Ki67 on regulatory T cells (Tregs), CD4+ and CD8+ T cells and in Treg frequencies and absolute eosinophil counts in most of the observed patients, while frequencies of nonclassical (CD16+) monocytes were significantly decreased at a later follow-up time point. However, neither dynamic alterations in Tregs nor mMDSCs correlated with patients' OS (but retained their prognostic capacity under therapy when the cohort was dichotomized according to their median frequencies at the respective time point). Interestingly, early increases of absolute lymphocyte counts and delayed increases of peripheral CD4+ and CD8+ T cell frequencies within the pool of lymphocytes were significantly associated with a better outcome of ICI {1 year survival rate: 93.3%, response rate [best overall response (BOR) following immune-related response criteria (irRC)]: 71.4%} (21). Next, we investigated, also in partially overlapping cohorts, patients' peripheral blood CD4+ and CD8+ T cell differentiation signatures and PD-1 expression because that population was previously found in melanoma to harbor a pool of clonally expanded, tumor-reactive cells (22, 23). We found that an immune-activated CD8+ T cell compartment, characterized by higher frequencies of CD8+ effector memory type 1 (EM1) cells (CD45RA– CCR7– CD27+ CD28+) and lower frequencies of CD8+ T_EMRA_ cells (CD45RA+ CCR7– CD27– CD28–) before starting CTLA-4 blockade correlated significantly with a more favorable outcome in univariate analyses (1 year survival rates: 46.4 vs. 35.4% for high vs. low CD8+ EM1 cells; 46.7 vs. 35% for low vs. high CD8+ T_EMRA_ cells). Interestingly, the frequency of PD-1 expression on peripheral CD8+ EM1 cells was not informative for therapy outcome at baseline, but a decrease of this population during therapy correlated with an improved clinical response (BOR following irRC) (24). However, due to limited sample material, we did not have the opportunity to investigate whether PD1+ EM1 CD8+ T cells that recognized tumor antigens increased during therapy in responding metastases. We also do not know whether this population harbored (clonally expanded) tumor-reactive cells nor whether such cells, if present, might have been dysfunctional. Reading et al., provide a detailed discussion of the role of CD8+ memory T cells in tumor immunity in this context (25).

Investigations of γδ T cells revealed that these cells also possessed value as biomarker candidates for the outcome of ipilimumab therapy. We found higher peripheral frequencies of Vδ1+ and lower frequencies of Vδ2+ cells in stage IV patients before start of therapy than in an age- and sex- matched control cohort of healthy subjects; this effect was even more pronounced in short-term survivors (<9 months OS). In line with these findings, low Vδ1+ and high Vδ2+ T cell frequencies prior to therapy start correlated significantly in a univariate analysis with prolonged OS under therapy (1 year survival rates: 53.3 vs. 37.9% for low/high Vδ1+ and 54.2 vs. 39% for high/low Vδ2+) (13). Further investigation of the predictive capacity but also the functionality of γδ T cells under single-agent PD-1 treatment or in combination with CTLA-4 inhibitory therapies is currently ongoing under the aegis of the German Research Unit 2799 (Receiving and Translating Signals via the γδ T cell receptor; https://for2799.de/). In that context, it is important to be aware of potential pitfalls in the characterization of circulating and tissue-resident γδ T cells because the application of commercially available reagents to classify these unconventional T cells is not always trouble-free. Based on the published literature and our own experience, we have recently provided an overview of how such pitfalls might be circumvented and suggested basic requirements for harmonization and standardization of γδ T cell immunomonitoring approaches (26).

## Peripheral Biomarkers for Melanoma With Pembrolizumab Treatment

We have recently extended some of the above analyses to melanoma patients treated with single agent anti-PD-1 antibodies and investigated routine baseline blood parameters and clinical meta-data in a multi-center study before starting anti-PD-1 blockade. High relative eosinophil counts, relative lymphocyte counts, low serum LDH-levels and the absence of metastasis in other than soft-tissue/lung were independent baseline characteristics that associated with favorable OS. The more of these favorable baseline factors were evident in a given patient, the better was his/her survival probability (1 year survival rates: 83.9% for best factor combination; 14.7% for the poor factor combination) (27).

In a recent study from Bochem et al. (28), we investigated peripheral blood T-cell phenotypes, searching for biomarker candidates predicting treatment outcome in melanoma patients under PD-1 inhibition. Patients with lower than median frequency of peripheral PD-1+ CD56+ T-cells had a significantly longer OS (1 year survival rate 78.4 vs. 52.8% for low vs. high frequencies), progression free survival (1 year progression-free survival rate 35.1 vs. 27.8% for low vs. high frequencies) and superior clinical benefit (59.5 vs. 27.8% for low vs. high frequencies; BOR following RECIST 1.1 criteria) compared to the reciprocal group. Interestingly, neither frequencies of “classical” CD56– CD4+ nor CD56– CD8+ T-cells, nor of the PD-1+ population within the CD4 or CD8 subsets was associated with clinical outcome (28). Only little is known about PD-1+ CD56+ T-cells in human cancers. Thus, future investigations are required for a better characterization of this heterogeneous cell population that presumably comprises large fractions of “non-classical” T cells, like NKT-like cells or γδ T cells.

To overcome limitations in the PD-1 detection in sample material obtained from patients under PD-1 therapy, we found it important to employ an experimental protocol to deal with steric hindrance between still-bound therapeutic antibodies and competition with the diagnostic antibody. This might be the reason why accurate PD-1 quantification in such samples has been problematic. Saturation of the patient's T cells with the therapeutic PD-1 antibody followed by secondary detection of the latter was necessary to allow accurate quantification of PD-1 on the cell surface (29).

## Peripheral-vs.-Tissue Biomarkers for Breast Cancer

To investigate whether other solid cancers behave similarly to melanoma in terms of the prognostic and predictive value of peripheral immune biomarkers, we elected to study breast cancer. We had already shown many years ago that Her2/neu peptides 776–788 and 884–899 were naturally-processed and presented TAA (30, 31). Due to our interest in the impact of age and immunosenescence on cancer immunity, we elected to study newly-diagnosed older women and found that the ability of patient's PBMCs to respond to TAA *in vitro*, in this case to her2/neu peptides, was also informative for breast cancer (32). Results paralleled findings in melanoma, demonstrating that prognostic impact depended on the pro- anti-inflammatory cytokine balance in the responding T cells (33). Moreover, the main markers in peripheral blood, namely, levels of mMDSCs, were also important indicators of survival in breast cancer as well as melanoma, and a combination of mMDSC levels and her2-reactivity even more so (32), as was the level of circulating plasmacytoid dendritic cells (34). It may be clinically important to note that cell surface marker immune phenotyping in older breast cancer patients identified correlations between baseline immune profile and geriatric assessment (35). Thus, frailer patients had higher levels of granulocytic cells but lower levels of cells with suppressor phenotypes including mMDSCs and Tregs, with none of these immune populations correlating with chronological age, but rather with frailty itself. The implications of these findings remain to be clarified, but clearly suggest that immune signatures correlating with clinical outcome depend on the physical state of the patient and can (in the case of elderly patients) be partly identified by geriatric frailty assessments (36). Whether the same is true for tumor-infiltrating immune cells in breast cancer is not yet established, but differential densities of CD8+ and CD163+ cells in the tumor core and margins were found to have significant prognostic value for survival (allowing better patient stratification than TNM staging, tumor size, lymph node invasion or histological grade). Patients having favorable immune signatures had favorable clinical outcomes despite poor clinicopathological parameters (37). These findings parallel many others in different cancers (38, 39). Of note in the light of our studies discussed above, low levels of intra-tumoral T cells and more granulocytic cells were present in clinically frail patients with shorter disease-specific survival (36). Together, these results are consistent with the notion that peripheral biomarkers are informative for clinically-relevant outcomes also in breast cancer, and may at least partially reflect what is seen in the tumor itself.

## Peripheral-vs.-Tissue Biomarkers for Renal Cancer

In renal cell carcinoma (RCC), expression of both PD-1 and PD-L1 within the primary tumor is associated with bad prognosis (40–42). In a recent study, we assessed the expression of five inhibitory receptors on T cells from RCC patients by flow cytometry (43). We found that PD-1, LAG-3 and Tim-3 were the three most upregulated checkpoint receptors on non-Treg CD4+ and CD8+ TILs as compared to autologous peripheral T cells, whereas PD-1, CTLA-4 and LAG-3 were dominant on tumor-associated Tregs. At the single cell level, PD-1 and LAG-3 were also the most often co-expressed receptors on CD4+ and CD8+ TILs. Still, there was a noticeable variability in the expression of the receptors between individuals, especially for LAG-3. Two main groups of tumors were identified. The first group (approximately half of the tumors, generally at more advanced T stages) was characterized by a high fraction of LAG-3+ T lymphocytes as well as other tumor-associated immune cells. A second group was constituted by tumors with rare expression of LAG-3 on all immune cell types. Our data are well in line with the results obtained by Giraldo et al., who showed that high densities of PD-1+ cells, and also of LAG-3+ cells, were associated with poorer prognosis in primary and metastatic RCC (40). PD-1 was slightly upregulated in peripheral T cells from RCC patients as compared to PBMCs from healthy donors, but for most other checkpoints, expression was only significantly increased in TILs, indicating that tumor-associated T cells, but not blood T cells, are more appropriate for checkpoint expression assessments.

In short-term functional experiments using RCC TILs activated with CD3 antibody in the presence of checkpoint-specific monoclonal antibodies, we found that simultaneous blocking of PD-1 and LAG-3 was more efficient in facilitating IFN-γ production than blocking of PD-1 alone or in combination with Tim-3. Here again, variability was observed between tumors. The frequency of IFN-γ producing CD8+ cells was increased ~2-fold for some patients, whereas it was nearly unchanged for others. This suggests that further parameters, possibly patient-specific, may be responsible for T cell unresponsiveness. Obviously, assessment of TIL functionality is technically challenging, and the development of simpler *in vitro* models could significantly improve testing. If successful, a following essential step would be to establish whether *in vitro* testing can readily predict clinical response to checkpoint blockade (43).

Whether checkpoint receptors (and their ligands) are expressed as similar levels in various tumors needs to be systematically addressed in middle to large scale patient cohorts. As an example, Li et al., recently showed that PD-1 is upregulated at comparable levels in TILs vs. PBMCs of eight different tumor types, including RCC (44). In contrast, Tim-3 expression was clearly lower in TILs from breast carcinoma, as compared to e.g., RCC or cervical cancer. Co-expression analysis of five inhibitory receptors also showed that some dominant combinations were observed on CD8+ T cells in most tumor types, whereas secondary patterns appear more tumor specific.

Note that the tumor digestion procedure in particular when enzymatic digestion is performed (45) but also the antibody clones and fluorochromes used [our unpublished observations and (26, 29, 46–48)] as well as the staining procedure (extra- or intracellular staining of CTLA-4) and the settings used for *in vitro* functional testing might all influence the analyses. Regarding *in vitro* functional analyses, different groups, including ourselves, have observed that the functional impact of the addition of blocking antibodies against checkpoint molecules is rather modest. Hence, here again, the field would certainly benefit from at least partial standardization of reagents and protocols, especially for flow or mass spectrometry multiparametric single cell studies, so that results obtained across various studies are more easily comparable.

## Conclusions and Perspectives

Although much effort is rightly being poured into analyzing the tumor microenvironment in order to understand the biology of cancer cell-host cell interactions, the routine application of such analyses for practical purposes is limited. While resected or biopsied tissue may also be useful for establishing baseline predictive biomarkers of response to therapy, monitoring of patient status at follow-up is challenging unless liquid biopsies can be employed. Using a minimally-invasive approach that can be repeated at will offers great advantages for immunomonitoring that may enable early detection of treatment response (or side effects) and enable therapies to be modified to replace ineffective treatments with others that might be more successful or tolerable. Combining immune biomarkers with routine clinical laboratory measures, as we have accomplished thus far and reviewed here, is merely an unsophisticated start to this effort, but possesses the advantage of feasibility for many groups in the field. Future work will be able to focus more closely on both tumor-derived and host-derived factors as determined in liquid biopsies. The former include circulating tumor cells (49), cell-free tumor DNA (50), exosomes containing tumor antigens (51), and soluble factors produced by the tumor; the latter include tumor antigen-specific T and B cells, innate immune cells and regulatory elements. Compound constellations of such markers will allow us to refine the clusters of parameters that we are beginning to find informative for monitoring cancer patients on immunotherapy (15, 21). Ideally, a blood-based “doctor's office” test would facilitate more rapid, safer and cheaper immune monitoring for therapy selection and modification.

## Author Contributions

KW-H, CG, and GP contributed jointly to conception and design of the study. GP wrote the first draft of the manuscript. KW-H and BW contributed the sections about the checkpoint blockade era in melanoma. GP wrote the sections discussing data from the pre-checkpoint era in melanoma and the section about breast cancer. CG contributed the discussion of renal cancer data. All authors contributed to manuscript revision, read, and approved the submitted version.

## Conflict of Interest

The authors declare that the research was conducted in the absence of any commercial or financial relationships that could be construed as a potential conflict of interest.
